# In elite athletes with meniscal injuries, always repair the lateral, think about the medial! A systematic review

**DOI:** 10.1007/s00167-022-07208-8

**Published:** 2022-11-02

**Authors:** Riccardo D’Ambrosi, Amit Meena, Akshya Raj, Nicola Ursino, Laura Mangiavini, Mirco Herbort, Christian Fink

**Affiliations:** 1 IRCCS Istituto Ortopedico Galeazzi, Milan, Italy; 2 Dipartimento di Scienze Biomediche per la Salute, Università degli Studi di Milano, Milan, Italy; 3 Gelenkpunkt-Sports and Joint Surgery, FIFA Medical Centre of Excellence, Innsbruck, Austria; 4 Research Unit for Orthopaedic Sports Medicine and Injury Prevention (OSMI), Medical Informatics and Technology, Private University for Health Sciences, Innsbruck, Austria; 5 Central Institute of Orthopaedics, Vardhman Mahavir Medical College and Safdarjung Hospital, New Delhi, India; 6 Orthopadische Chirurgie Munchen, Munchen, Germany

**Keywords:** Meniscal suture, Meniscal repair, Meniscectomy, Return to sport, Elite athletes, Professional

## Abstract

**Purpose:**

This study aimed to evaluate and compare the time required to return to sports (RTS) after surgery, the rate of revision surgery and the time required for RTS after revision surgery in elite athletes undergoing meniscal repair or partial meniscectomy, particularly analysing the difference between medial and lateral menisci. It was hypothesised that both procedures would entail similar, high rates of RTS, with the lateral meniscus exhibiting higher potential healing postprocedure compared to the medial meniscus.

**Methods:**

A systematic review was conducted based on the PRISMA guidelines. Quality assessment of the systematic review was performed using the AMSTAR-2 checklist. The following search terms were browsed in the title, abstract and keyword fields: ‘meniscus’ or ‘meniscal’ AND ‘tear,’ ‘injury’ or ‘lesion’ AND ‘professional,’ ‘elite’ or ‘high-level’ AND ‘athletes,’ ‘sports,’ ‘sportsman,’ ‘soccer,’ ‘basketball,’ ‘football’ or ‘handball’. The resulting measures extracted from the studies were the rate of RTS, level of RTS, complications, revision surgery and subsequent RTS, Tegner, International Knee Documentation Committee (IKDC) and Visual Analogue Scale (VAS).

**Results:**

In this study, the cohort consisted of 421 patients [415 (98.6%) men and 6 (1.4%) women] with a mean age of 23.0 ± 3.0 years. All patients were elite athletes in wrestling, baseball, soccer, rugby or handball. While 327 (77.7%) patients received partial meniscectomy at a mean age of 23.3 ± 2.6 years, 94 (22.3%) patients received meniscal repair at a mean age of 22.1 ± 4.0 years. After partial meniscectomy, 277 patients (84.7%) returned to their competitive sports activity and 256 (78.3%) returned to their pre-injury activity levels. A total of 12 (3.7%) patients required revision surgery because of persistent pain [5 (1.5%) patients], chondrolysis [2 (0.7%) patients] or both chondrolysis and lateral instability [5 (1.5%) patients]. Ten (83.3%) of the twelve patients had involvement of the lateral meniscus, whereas the location of injury was not specified in the remaining two patients. After revision surgery, all patients (100%) resumed sports activity. However, after meniscal repair, 80 (85.1%) athletes returned to their competitive sports activity and 71 (75.5%) returned to their pre-injury activity levels. A total of 16 (17.0%) patients required partial meniscectomy in cases of persistent pain or suture failure. Of these, 4 (25%) patients involved lateral and medial menisci each and 8 (50%) patients were not specified. After revision surgery, more than 80.0% of the patients (13) resumed sports activity.

**Conclusions:**

In elite athletes with isolated meniscal injury, partial meniscectomy and meniscal suture exhibited similar rates of RTS and return to pre-injury levels. Nonetheless, athletes required more time for RTS after meniscal repair and exhibited an increased rate of revision surgery associated with a reduced rate of RTS after the subsequent surgery. For lateral meniscus tears, meniscectomy was associated with a high rate of revision surgery and risk of chondrolysis, whereas partial medial meniscectomy allowed for rapid RTS but with the potential risk of developing knee osteoarthritis over the years. The findings of this systematic review suggested a suture on the lateral meniscus in elite athletes because of the high healing potential after the procedure, the reduced risk of developing chondrolysis and the high risk of revision surgery after partial meniscectomy. Furthermore, it is important to evaluate several factors while dealing with the medial meniscus. If rapid RTS activity is needed, a hyperselective meniscectomy is recommended; otherwise, a meniscal suture is recommended to avoid accelerated osteoarthritis.

**Level of evidence:**

Level IV.

**Study registration:**

PROSPERO-CRD42022351979 (https://www.crd.york.ac.uk/prospero/display_record.php?RecordID=351979).

## Introduction

Meniscal injuries constitute the majority of injuries to the knee, with more than 60% of knee injuries reported to be injuries to the menisci [1]. The overall incidence of meniscal injuries that need operative management is between 60 and 70 per 100,000 person-years [27]. Athletes are prone to knee injuries more than the general population, particularly those involved in contact and pivoting sports [38]. In athletes of contact sports such as football and basketball, meniscal tears were the most prevalent knee injury, occurring in nearly half of the study population of over 900 athletes in a systematic review by Flanigan et al. [11]. When the injured meniscus is operated upon, it is treated with either meniscal repair or meniscectomy, which is usually a partial meniscectomy. The decision for management is based on the patient profile and the characteristics of the tear, such as size, location and reducibility [12, 13]. Most surgeons prefer to repair the menisci wherever amenable, with repair being well suited for vertical or longitudinal tears in the red‒red or red‒white regions of the menisci, which are reducible [19]. A partial or complete meniscectomy can lead to the early occurrence of degenerative changes [7, 29]. Repair of meniscal tears has been believed to prevent or delay the onset of degenerative changes in the knee [3]. However, for an in-season player, a partial meniscectomy may be better in the short term, as return to play after a partial meniscectomy has been found to be faster than with repair, [5, 15, 16, 21] which would make all the difference to the careers of athletes at elite levels and the fortunes of their team. Recent literature has found the highest rate of return to sport in athletes who undergo a partial meniscectomy [22]. However, there is yet to be a definitive procedure of choice for the surgical management of meniscal injury in high-level athletes. The current systematic review was undertaken to perform a comprehensive analysis of the literature on the surgical management and outcomes of meniscal injury in elite athletes. The purpose of the study was to evaluate the return to sport (RTS) duration after surgery, rate of revision surgery and subsequent return to sport after revision in elite athletes undergoing meniscal repair or partial meniscectomy and to compare these outcomes between the two types of procedures, particularly analysing the difference between medial and lateral menisci. The hypothesis was that (1) for isolated meniscal injuries, both procedures would entail a similar, high return to sport and (2) the lateral meniscus would be associated with a higher healing potential than the medial meniscus after suture repair.

## Materials and methods

The current systematic review was performed following the Preferred Reporting Items for Systematic Reviews and Meta-Analyses (PRISMA) guidelines and is registered in the PROSPERO Registry CRD42022351979 (https://www.crd.york.ac.uk/prospero/display_record.php?RecordID=351979) [28, 33]. The AMSTAR-2 checklist was used to confirm the quality of the systematic review (Appendices 1 and 2) [32].

### Eligibility criteria

The literature selected for this study was based on the following criteria.

#### Study design

Randomised controlled trials (RCTs), controlled (nonrandomised) clinical trials (CCTs), prospective and retrospective comparative cohort studies, case‒control studies and case series were included. Case reports and case series that did not report data on return to sports were excluded.

#### Participants

Studies conducted on skeletally mature elite athletes treated surgically for isolated meniscal lesions (either medial or lateral) with partial meniscectomy or meniscal suture and evaluated for return to sports activity. Concomitant anterior cruciate ligament (ACL) reconstruction was considered an exclusion criterion.

#### Interventions

Studies that reported data on return to sports activity and level of return in elite athletes treated surgically for isolated meniscal lesions with partial meniscectomy or meniscal repair.

For meniscal treatment, the surgical technique (percentage of meniscus involved, type of lesion, system for suture) and rehabilitation protocol were collected. Concomitant anterior cruciate ligament (ACL) reconstruction was considered an exclusion criterion.

#### Types of outcome measures

The outcome measures extracted from the studies were rate of return to sport, level of return, complications, revision surgery and subsequent return to sport, Tegner, IKDC and VAS.

Meniscal lesions were classified according to the International Society of Arthroscopy, Knee Surgery and Orthopaedic Sports Medicine (ISAKOS) classification [4].

### Information sources and search

A systematic search for relevant literature was performed in the PubMed (MEDLINE), Scopus, EMBASE and Cochrane Library databases of all studies published in English from January 1990 to July 2022. The publication date was not considered an inclusion criterion. The search was carried out in July 2022. Two independent reviewers (RD and AM) assisted in conducting and validating the search. The following search terms were entered into the title, abstract, and keyword fields: “meniscus” or “meniscal” AND “tear” or “injury” or “lesion” AND “professional” or “elite” or “high-level” AND “athletes” or “sports” or “sportsman” or “soccer” or “basketball” or “football” or “handball”. Finally, only papers published in English were included.

### Data collection and analysis

#### Study selection

The retrieved articles were first screened by title and, if found relevant, screened further by reading the abstract. After excluding studies not meeting the eligibility criteria, the entire content of the remaining articles was evaluated for eligibility. To minimise the risk of bias, the authors reviewed and discussed all the selected articles, references, and articles excluded from the study. In case of any disagreement between the reviewers, the senior investigator made the final decision. At the end of the process, further studies that might have been missed were manually searched by going through the reference lists of the included studies and relevant systematic reviews.

#### Data collection process

The data were extracted from the selected articles by the first two authors using a computerised tool created with Microsoft Access (Version 2010, Microsoft Corp, Redmond Washington). Each article was validated again by the first author before analysis. For each study, data regarding the patients were extracted (age, sex, sports practised), their injuries (type, aetiology), the surgical technique, rehabilitation protocol, return to sport, level of post-operative activity, rate of complications, new surgeries, return to sport after revision surgery and clinical outcomes.

#### Level of evidence

The Oxford Levels of Evidence set by the Oxford Centre for Evidence-Based Medicine were used to categorise the level of evidence [6].

#### Evaluation of the quality of studies

The quality of the selected studies was evaluated using the Methodological Index for Nonrandomized Studies (MINORS) score [34]. The checklist includes 12 items, of which the last four are specific to comparative studies. Each item was given a score of 0–2 points. The ideal score was set at 16 points for noncomparative studies and 24 for comparative studies.

Furthermore, according to AMSTAR-2 guidelines, every article was assessed using the ROBINS-I tool [14, 32].

## Results

### Search results

**Fig. 1:**
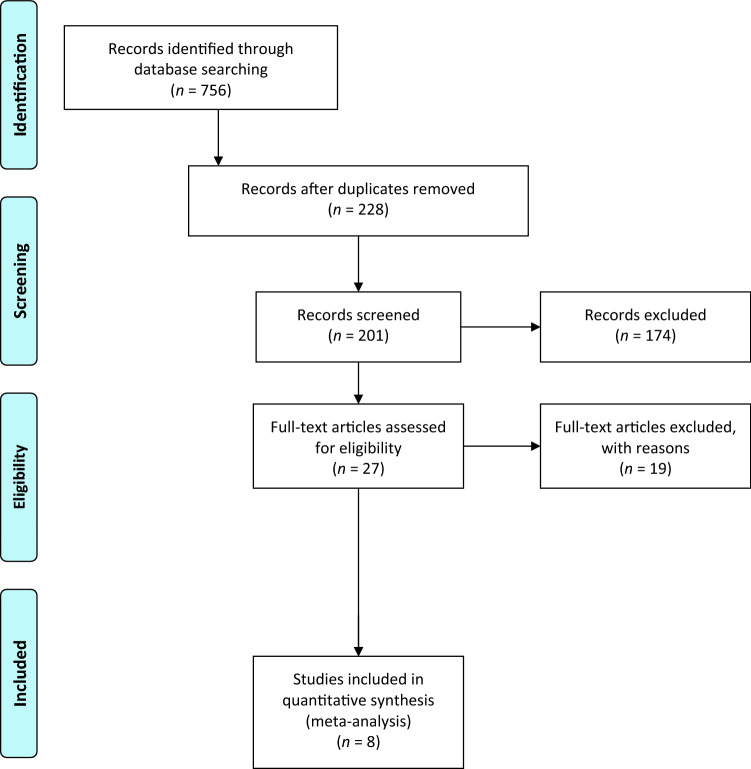
A flowchart of the literature screening performed in this study

The electronic search yielded 756 studies. After 528 duplicates were removed, 228 studies remained, of which 201 were excluded after reviewing the abstracts, bringing the number down to 27. An additional 19 articles were excluded based on the aforementioned inclusion and exclusion criteria. No additional studies were found by manually searching the reference lists of the selected articles. This left 8 studies for analysis. Figure 1 shows the flowchart depicting the selection process for studies [2, 9, 10, 23, 24, 26, 35, 36]. The analysed studies had a mean MINORS score of 8.5 (median 9; range, 7–10), which confirmed the methodological quality of the available literature (Table 1).

**Table 1:** Characteristics of the selected studies

Author, year	MINORS	Level of evidence	Patients	Gender (M:F)	Treatment	Age	Sports
Marigi, 2021 [24]	10	4	85 (56 partial meniscectomy/29 meniscal repair)	85:0	Partial Meniscectomy (56)/Meniscal Repair (29)	Meniscectomy: 18.0 ± 2.8 Meniscal Repair: 16.7 ± 2.9	Wrestling
Erickson, 2022 [10]	9	3	31	31:0	Meniscal repair	24.0 ± 3.0	Baseball
Erickson, 2022 [9]	9	3	168	168:0	Partial meniscectomy	25.0 ± 5.0	Baseball
Alvarez-Diaz, 2016^a^ [2]	9	4	14	14:0	Meniscal repair	28 (18–37)	Soccer
Mariani, 2008 [23]	7	4	5	5:0	Partial meniscectomy	26.8	Soccer
Nawabi, 2014 [26]	9	3	90	90:0	Partial meniscectomy	Lateral (42): 23.7 ± 4.1 Medial (48): 22.4 ± 3.6 years	Soccer
Sonnery-Cottet, 2014 [35]	7	4	8 patients (10 knees)	7:1	Partial meniscectomy	25.2	2 Rugby 3 Handball 3 Football
Tucciarone, 2012^a^ [36]	8	3	20	15:5	Meniscal repair	23 (18–26)	Soccer or Basketball

^a^Only isolated meniscal repair group has been considered

### Patient and study characteristics

Table 1 shows the characteristics of the cohorts involved in the 8 selected studies and a summary of their data. All studies were level 3 [9, 10, 26, 36] (4–50%) or 4 [2, 23, 24, 35] (4–50%).

The cohort of patients consisted of 421 participants [415 (98.6%) men and 6 (1.4%) women] with a mean age of 23.0 ± 3.0 years. All patients were elite athletes involved in wrestling, baseball, soccer, rugby or handball.

The mean age of the 327 (77.7%) patients who received a partial meniscectomy was 23.3 ± 2.6 years; 326 (99.7%) of them were men and 1 (0.3%) was a woman.

Of the 94 (22.3%) patients who received meniscal repair, 89 (94.7%) were men and 5 (5.3%) were women, with a mean age of 22.1 ± 4.0 years.

### Partial meniscectomy

#### Surgical details

In partial meniscectomy, 329 menisci were treated in 327 patients, of which 166 (50.5%) were medial menisci, 147 (44.6%) lateral menisci and both in 16 (4.9%) patients. The lesions were classified in only 3 studies, for a total of 151 tears, as follows: 9 (6.0%) simple, 18 (11.9%) bucket-handle, 71 (47.0%) complex, 18 (11.9%) radial, 5 (3.3%) horizontal, 1 (0.7%) flap and 29 (19.2%) vertical. Surgical details are reported in Table 2.

**Table 2:** Surgical and rehabilitation protocol

Lead author	Type of lesion	Type of tear	Chronicity of injury	Type of treatment	Rehabilitation protocol
Marigi, 2021 [24]	Meniscectomy: 40 medial 15 lateral 1 both Repair: 14 medial 14 lateral 1 both	Meniscectomy: 9 simple 18 bucket-handle 29 complex Repair: 15 simple 10 bucket- handle 4 complex	Meniscectomy: 42 acute 14 chronic Repair: 20 acute 9 chronic	Meniscectomy: 56 Meniscal Repair: 3 bioabsorbable arrows 10 all-inside 10 inside-out 3 root repair 3 inside-out + all-inside	
Erickson, 2022 [10]	7 (28%) medial 18 (72%) lateral			15 (60%) all-inside 3 (12%) inside-out 3 (12%) outside-in 1 (4%) root 3 (12%) combined	
Erickson, 2022 [9]	78 (46%) medial 75 (45%) lateral 15 (9%) both		101 (70%) acute 43 (30%) overuse	Partial meniscectomy	
Alvarez-Diaz, 2016^a^ [2]		All complete and longitudinal tears in the posterior horn		Meniscal suture all-inside with FastFix system	Active exercise the day after surgery Partial weight-bearing for 4 weeks
Mariani, 2008 [23]	5 lateral	3 radial 1 horizontal 1 flap		Partial meniscectomy	
Nawabi, 2014 [26]	42 lateral 48 medial	Lateral: 5 vertical 4 horizontal 12 radial 21complex Medial: 24 vertical 0 horizontal 3 radial 21 complex		Partial meniscectomy: Percentage of meniscal excision: Lateral: 19.2 (10–30) Medial: 27.6 (15–60)	
Sonnery-Cottet, 2014 [35]	10 lateral	n.a	Traumatic tears		
Tucciarone, 2012^a^ [36]	10 medial 10 lateral	20 Body-Posterior horn 18 Longitudinal vertical—2 Bucket handle		Meniscal suture all-inside with FastFix system	No WB for 4 weeks Partial WB 4–6 weeks Complete WB from VII week

*WB* weight-bearing

^a^Only isolated meniscal repair group has been considered

#### Return to sport

Table 3 shows the return to sports of the cohorts involved in the 8 selected studies and a summary of their data. After the first surgery, 277 patients (84.7%) returned to their competitive sports activity and 256 (78.3%) returned to the same pre-injury activity level.

**Table 3:** Return to sports rate, complications, revision surgeries and clinical and functional outcomes

Lead author	Time to return	Return to sport	Level of return	Complications	New surgeries	Return to sport for patients that required new surgeries	Pre-operative score	Post-operative score
Marigi, 2021 [24]		76 (89%) returned to sports No differences between the two groups	55 (65%) pre-injury level No differences between the two groups	8 (9.4%) persistent pain	All for revision surgery: 2 (3%) after meniscectomy 6 (21%) after meniscal repair	100% return to sport 82% return to wrestling	Tegner 6.5 (5.9–7.2) VAS 4.6 (4.1–5.1) IKDC 67.3 (64.1–70.6)	Tegner 8.3 (8.0–8.6)^a^ VAS 0.54 (0.35–0.73)^a^ IKDC 96.0 (94.5–97.5)^a^
Erickson, 2022 [10]	209 ± 84 days	23 (74%) returned to sports	21 (68%) same or higher level 2 (6%) lower level 8 (26%) did not return		6 (19)% had failed repair and underwent subsequent meniscectomy on the same knee 4 (12%) medial meniscus 2 (6%) lateral meniscus	5 (83%) returned at the same level		
Erickson, 2022 [9]	0–30 days 9% 30–60 days 20% 60–90 days 7% 90–120 days 6% 120–150 days 5% 150–180 days 11% 210–240 days 10% > 240 days 21%	134 (80%) return to sport	127 (76%) return to same or higher level 7 (4%) return to lower level 34 (20%) did not return		0			
Alvarez-Diaz, 2016^b^ [2]	129 days	Immediate: 13 (92%) return to sport	Immediate: 13 (92%) return same level	6 (21.4%) post-operative pain	2 (6.7%) partial meniscectomy	2 (100%) return to same level	Tegner pre-injury 9 (9–10)	Tegner last follow-up 6 (6–9)
Mariani, 2008 [23]		0 (0%)	0 (0%)	5 (100%) chondrolysis + posterolateral instability	5 (100%) lateral revision surgery of which: 1 microfracture + popliteus tendon augmentation 4 capsular tensioning	5 (100%) return same level		
Nawabi, 2014 [26]	Lateral: 49 days Medial: 35 days^c^	87 (96.7%)	87 (96.7%) return to their activity	Lateral: 29 (69%) effusion and pain Medial: 4 (8%) fat-pad inflammation	3 (3.3%) lateral further arthroscopy for persistent pain	3 (100%) return to same level		
Sonnery-Cottet, 2014 [35]	218 days	6 (75%)	6 (75%) same level	8 (100%) pain and swelling with chondrolysis	2 (25%) lateral needed for arthroscopic lavage	2 (100%) return same level		IKDC 82.64 ± 8.61 Lysholm 86.6 ± 6.44 Tegner 9 ± 1.41
Tucciarone, 2012^b^ [36]		18 (90%)	18 (90%) same level	2 (10%) persistent pain for meniscal flap and not healing	2 (10%) lateral partial meniscectomy			

*VAS* visual analogue scale for pain, *IKDC* international knee documentation committee

^a^Statistical significant difference between the two time points

^b^Only isolated meniscal repair group has been considered

^c^Statistical significant difference between the two groups

#### Time to return to sports

The meniscectomy time to return to sports ranged from a small cohort of patients who returned after less than 30 days [9] to a mean of 35–50 days. In the study published by Sonnery-Cottet, the mean time to return to sports was 218 days in patients treated without revision surgery for chondrolysis [35]. A significant difference was noted in the study of Nawabi et al. between lateral and medial meniscus (49 days versus 35 days; *p* < 0.05) [26].

#### Revision surgery and subsequent return to sport

A total of 12 (3.7%) patients required revision surgery due to persistent pain in 5 (1.5%) patients, chondrolysis in 2 (0.7%) patients, and chondrolysis and lateral instability in 5 (1.5%) patients.

Of these, ten out of twelve (83.3%) involved the lateral meniscus, while in the remaining patients, they were not specified. After revision surgery, all patients (100%) resumed sports activity.

### Meniscal repair

#### Surgical details

Of the 88 procedures analysed in meniscal repair, 31 (35.3%) were treated medially, 42 (47.7%) were treated laterally and 1 (1.1%) was treated for both tears, while in 14 (15.9%) patients, these parameters were not specified. The lesions were classified into 63 patients as follows: 15 (23.8%) simple, 12 (19.1%) bucket-handle, 4 (6.3%) complex, 14 (22.2%) longitudinal and 18 (28.6%) longitudinal/vertical. For the repair, 3 (3.4%) were performed with bioabsorbable arrows, 59 (67.1%) all-inside, 13 (14.8%) inside-out, 4 (4.5%) root repair, 6 (6.8%) combined and 3 (3.4%) outside-in. Surgical details are reported in Table 2.

#### Return to sport

After the first surgery of meniscal repair, 80 (85.1%) of the athletes returned to their competitive sports activity and 71 (75.5%) returned to the same pre-injury activity level.

#### Time to return to sports

After meniscal suture, the mean time to return to sports ranged from 129 to 209 days (mean 184.1 days) [2, 10].

#### Revision surgery and subsequent return to sport

After meniscal repair, 16 (17.0%) patients required a partial meniscectomy in all patients for persistent pain or suture failure.

Of these, 4/16 (25%) involved the lateral meniscus, 4/16 (25%) involved the medial menisci and 8 (50%) patients were not specified. After revision surgery, more than 80.0% of the patients (13) resumed sports activity.

## Discussion

The most important finding of the current systematic review was that the rate of return to competitive sports was similar for both meniscal repair and partial meniscectomy, and similar rates were found for return to the pre-injury level of sports activity. However, there was a shorter time for return to sport with meniscectomy compared to meniscal suture repair and a higher level of revision surgeries after meniscal repair, with a lower rate of return to sport after revision for failed meniscal repair. In particular, a very high percentage of revision surgery was required for lateral meniscus (> 80%) after partial meniscectomy, while for meniscal repair, data are poorly evaluated because in 50% of the failures, it was not specified whether it was medial or lateral meniscus but certainly compared with meniscectomy, there is a higher failure rate in the medial meniscus and a lower failure rate in the lateral.

The systematic review by Eberbach et al. assessed the return to sport (RTS) after isolated medial meniscus repair. Their review found a return to sports activity rate of 89%, which is similar to the current study [7].

Lee et al. analysed return to sports in high-level athletes after either partial meniscectomy, meniscal repair or a meniscal allograft transplant. They found that the shortest time to return to sports was with partial meniscectomy, and the highest rate of return to sports was again with partial meniscectomy (up to 100%). However, the rate of RTS after meniscal repair was 81–88.9% in the studies the authors analysed, which is in keeping with what the current review found [22]. An earlier systematic review by Ekhtiari et al. had findings similar to those of the abovementioned study, with players returning to sports significantly earlier after a partial meniscectomy and a similarly high proportion of players returning to pre-injury levels, both after repair and after partial meniscectomy [8].

A more recent systematic review by Blanchard et al. also found similar rates of return to sport activity following isolated medial meniscus repairs, of 83.1%. However, in comparison to the current study, those authors found a higher rate of revision following medial meniscal repair, at 12.4% [3].

The mean time to return to sport was lower with meniscectomy in the current review. Eberbach et al. reported in their systematic review on the mean time to RTS after isolated medial meniscal repair [7]. They found the mean RTS to be 8.7 months, which was greater than the finding in the current study, which ranged from approximately 3–7 months. These results are consistent with those found in previous research by Lee et al. [22] and Ekhtiari et al. [8], both systematic reviews indicating faster RTS following meniscectomies.

However, the type of surgery on the injured meniscus may only be one of multiple factors with bearing on time to RTS. The meniscus that is injured may also influence RTS, as it was found to have a greater time of resumption of sports with lateral meniscal injuries compared with medial meniscal injuries [26]. There have also been reports of a greater incidence of pain, effusions and joint line tenderness after partial lateral meniscectomy [18, 26].

Recent studies indicate that the lateral meniscus has better healing potential with a lower revision rate following repairs [30]. This was reported by Rönnblad et al. in their dual-centre retrospective case–control study [30]. According to the authors, this tendency is conferred by the greater mobility of the lateral meniscus compared to the more firm attachment of the medial meniscus to the tibial plateau [30]. Similarly, the systematic review by Schweizer et al. found a lower failure rate with lateral meniscus (19.5%) compared to medial meniscal repairs (24.4%) [31]. In the current study, most of the surgical sutures were performed using an all-inside technique (> 65.0%); this does not affect the final result. In fact, in 2019, Kang et al. investigated arthroscopic meniscus repair by comparing all-inside and inside-out suture techniques, confirming that both techniques are equally effective in terms of clinical outcomes and failure rate [17].

With respect to revision surgeries after partial meniscectomy, although a greater number were found with lateral meniscus, concomitant chondrolysis and chondrolysis with lateral instability were also found. These confounding factors need to be taken into account when interpreting the results of the current study regarding postmeniscectomy revision surgery, as a knee with lateral instability will understandably have greater stresses on the lateral compartment and will result in greater damage to the lateral meniscus. Similar results were also reported by Nawabi et al., who found a longer time for return to play after lateral meniscectomy than after medial meniscectomy in elite professional soccer athletes [26].

This is one of the first systematic reviews that has analysed return to sports in high-level elite athletes in multiple sporting disciplines after either meniscal repair or partial meniscectomy and, to the best of the authors’ knowledge, the first systematic review to analyse return to sport after revision surgery for a meniscal injury. The strengths of the current study are a large study population, inclusion of only elite level athletes, inclusion of multiple disciplines of sport and the analysis of return to sport after revision surgery. Previous systematic reviews on the current topic had either a small study population, included only a single sport, or included no information on return to sport after revision surgery.

This study exhibits several limitations. First, all studies were categorised as level 3 or 4, which prevented a meta-analysis [25] of the results. Moreover, the results of sutures and meniscectomy were simultaneously analysed in only one study [24]. Second, several studies did not report all the information regarding the type of treatment, type and location of the lesion, type of suture and failure analysis.

The findings of this systematic review and the current literature suggested a suture on the lateral meniscus (the technique is indifferent) in elite athletes because of the high healing potential after the procedure, the reduced risk of developing chondrolysis and the high risk of revision surgery after partial meniscectomy. Furthermore, it is important to evaluate several factors while dealing with the medial meniscus. If rapid RTS activity is needed, a hyperselective meniscectomy is recommended; otherwise, a meniscal suture is recommended to avoid accelerated osteoarthritis, considering that meniscal suture exhibits a high probability of failure owing to the low reparative ability of the medial meniscus. Therefore, it was suggested that all of the abovementioned factors must be evaluated before deciding on surgically addressing a meniscal injury of an elite athlete (Table 4).

**Table 4:** Indications, pros, and cons in the treatment of isolated meniscus injuries in professional athletes according to location

Meniscus injury in elite athletes			
Type of lesions	Type of treatment	Pros	Cons
Medial	Meniscectomy	Fast return to sport [9, 26] Low revision rate [9, 26]	Risk of developing OA [20]
	Meniscal repair	OA prevention [37]	High risk of failure [10, 30, 31] Slower return to sport compared to meniscectomy [2, 10] Lower healing potential compared to lateral meniscal repair [30, 31]
Lateral	Meniscectomy	Fast return to sport [9, 26]	Risk of developing OA [37] Risk of chondrolysis [23, 35] High risk of revision surgery [23, 35]
	Meniscal repair	OA prevention [37] Higher healing potential compared to medial meniscus repair [30, 31]	Lower risk of revision surgery compared to medial meniscal suture [30, 31] Slower return to sport than meniscectomy [9, 10, 26]

*OA* osteoarthritis

## Conclusions

In elite athletes with isolated meniscal injury, partial meniscectomy and meniscal suture exhibited similar rates of RTS and return to pre-injury levels. Nonetheless, athletes required more time for RTS after meniscal repair and exhibited an increased rate of revision surgery associated with a reduced rate of RTS after the subsequent surgery. For lateral meniscus tears, meniscectomy was associated with a high rate of revision surgery and risk of chondrolysis, whereas partial medial meniscectomy allowed for rapid RTS but with the potential risk of developing knee osteoarthritis over the years.
